# Symptom prevalence and secondary attack rate of SARS‐CoV‐2 in rural Kenyan households: A prospective cohort study

**DOI:** 10.1111/irv.13185

**Published:** 2023-09-26

**Authors:** Katherine E. Gallagher, Joyce Nyiro, Charles N. Agoti, Eric Maitha, James Nyagwange, Angela Karani, Christian Bottomley, Nickson Murunga, George Githinji, Martin Mutunga, Lynette Isabella Ochola‐Oyier, Ivy Kombe, Amek Nyaguara, E. Wangeci Kagucia, George Warimwe, Ambrose Agweyu, Benjamin Tsofa, Philip Bejon, J. Anthony G. Scott, David James Nokes

**Affiliations:** ^1^ KEMRI‐Wellcome Trust Research Programme (KWTRP) Kilifi Kenya; ^2^ Department of Infectious Diseases Epidemiology London School of Hygiene and Tropical Medicine London UK; ^3^ Ministry of Health Government of Kenya Nairobi Kenya; ^4^ Nuffield Department of Medicine Oxford University Oxford UK; ^5^ School of Life Sciences and the Zeeman Institute for Systems Biology & Infectious Disease Epidemiology Research (SBIDER) University of Warwick Coventry UK

**Keywords:** household transmission, longitudinal cohort, SARS‐CoV‐2, seroprevalence

## Abstract

**Background:**

We estimated the secondary attack rate of SARS‐CoV‐2 among household contacts of PCR‐confirmed cases of COVID‐19 in rural Kenya and analysed risk factors for transmission.

**Methods:**

We enrolled incident PCR‐confirmed cases and their household members. At baseline, a questionnaire, a blood sample, and naso‐oropharyngeal swabs were collected. Household members were followed 4, 7, 10, 14, 21 and 28 days after the date of the first PCR‐positive in the household; naso‐oropharyngeal swabs were collected at each visit and used to define secondary cases. Blood samples were collected every 1–2 weeks. Symptoms were collected in a daily symptom diary. We used binomial regression to estimate secondary attack rates and survival analysis to analyse risk factors for transmission.

**Results:**

A total of 119 households with at least one positive household member were enrolled between October 2020 and September 2022, comprising 503 household members; 226 remained in follow‐up at day 14 (45%). A total of 43 secondary cases arose within 14 days of identification of the primary case, and 81 household members remained negative. The 7‐day secondary attack rate was 4% (95% CI 1%–10%), the 14‐day secondary attack rate was 28% (95% CI 17%–40%). Of 38 secondary cases with data, eight reported symptoms (21%, 95% CI 8%–34%). Antibody to SARS‐CoV‐2 spike protein at enrolment was not associated with risk of becoming a secondary case.

**Conclusion:**

Households in our setting experienced a lower 7‐day attack rate than a recent meta‐analysis indicated as the global average (23%–43% depending on variant), and infection is mostly asymptomatic in our setting.

## 1 BACKGROUND

Basic epidemiological parameters for the severe acute respiratory syndrome coronavirus‐2 (SARS‐CoV‐2) are still largely unknown for most African settings, for example, the basic reproduction number and ^1^, ^2^ infectious period and serial interval.^3^, ^4^ Data on these parameters and the virus's propensity to spread and cause coronavirus disease 2019 (COVID‐19) may help explain the differential burden of hospitalisations and deaths in Kenya compared with other settings, for example, South Africa or Europe.^5^

A systematic review of published articles up to March 2022 identified 135 household transmission studies of SARS‐CoV‐2.^6^, ^7^ Pooled secondary attack rates across studies were highest for the Omicron variant (43%, 95% CI 35.4–50.4) and then Alpha (36.4%, 95% CI 33.4–39.5), Delta (29.7%, 95% CI 23.0–37.3), and Beta (22.5%, 95% CI 18.6–26.8). Higher rates of transmission were documented from unvaccinated cases compared with vaccinated primary cases in four studies during waves of Alpha and Delta variants. However, follow‐up time across studies varied, the majority of these studies relied on viral testing of primary cases and contacts at a single timepoint; only a minority of studies involved prospective longitudinal follow‐up; very few integrated serological data and the majority only tested contacts of symptomatic cases.^6^, ^7^ Five studies included virus genome sequencing data to define probable transmission chains within the households.^8^, ^9^, ^10^, ^11^, ^12^ Only one study in the review was conducted on the African continent, in South Africa^13^ and to our knowledge, there have been no further published studies from the African continent since then.

The South African study found a 14‐day secondary attack rate within households of 24%, irrespective of symptoms of the primary case. Primary cases who were children, had high viral load, and had Beta or Delta viral variants (compared with Alpha) were more likely to be the source of onwards transmission. In total, 85% of infections were asymptomatic.^13^

A recent systematic review of six studies in the African region estimated 47% (95% CI 22%–74%) of infections were asymptomatic, in contrast to 24% in Europe (95% CI 17%–33%).^14^ Additionally, asymptomatic infections were less likely to result in onwards transmission (RR 0.32 95%I 16–64).^14^ However, the prevalence of asymptomatic infections is likely to be underestimated by the inclusion of studies where enrolment was dependent on participants presenting for testing.

Further data on the epidemiology in the African region are needed, and we aimed to estimate the secondary attack rate of SARS‐CoV‐2, the prevalence of symptoms, and identify risk factors for transmission among household contacts of PCR‐confirmed cases in rural Kenya.

## 2 METHODS

### 2.1 Study setting

The study took place within the Kilifi Health and Demographic Surveillance System (KHDSS), Kilifi County, Kenya.^15^ As of 8th April 2020, members of the community could report via a telephone hotline if they suspected they were infected with SARS‐CoV‐2; they were then traced by the county rapid response team (RRT) and tested within 1–3 days. Close contacts (defined by the RRT as face‐to‐face contact within 1 m for more than 15 min) of RT‐PCR‐confirmed cases were also traced by the RRT and a respiratory sample was collected for laboratory RT‐PCR testing at KEMRI‐Wellcome Trust Research Programme (KWTRP). Initially, the RRT prioritised sampling symptomatic cases or contacts, but, over time, both symptomatic and asymptomatic individuals were sampled. The study team accompanied the RRT and approached the individuals identified by the RRT and their households to consent for this study.

### 2.2 Study participants

The protocol was adapted from the World Health Organization UNITY household study protocols.^16^ Individuals identified by the RRT, that is, suspected index cases and the contacts of cases, were approached for enrolment on the study alongside the members of their household (defined as anybody who shared the same cooking space). Individuals with respiratory illness who visited three outpatient facilities under surveillance and tested positive for SARS‐CoV‐2 were also recruited with their households. Contacts of cases and their households were included in an effort to start study follow‐up as early as possible in the transmission chain within the household. A household was eligible for enrolment if it consisted of two or more people and was accessible by vehicle and if written permission to engage with household members about the study was granted by the household head. Written informed consent was obtained from each household member aged ≥18 years. Parents/guardians were asked to provide written informed consent for any individual less than 18 years of age. Children aged 13–17 were asked to provide written assent.

### 2.3 Study procedures

**FIGURE 1:**
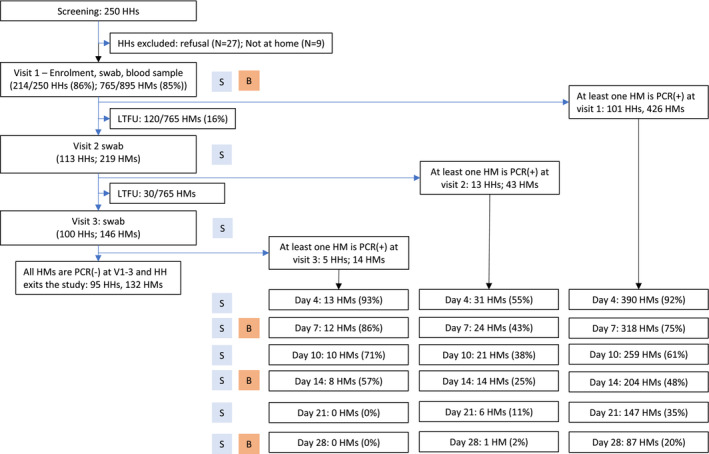
Participant flow. Abbreviations: HH: household; HM: household member; S: naso‐oropharyngeal swabs; PCR(+): a positive polymerase chain reaction assay results indicating presence of SARS‐CoV‐2; PCR(−) a negative polymerase chain reaction assay result indicating no evidence of SARS‐CoV‐2. Key: B = blood sample; S = nasopharyngeal swabs; *N* = number of households; *n* = number of individuals (household members).

At baseline, a questionnaire, a blood sample (3–5 mL), and naso‐oropharyngeal swabs were collected from all consenting household members (regardless of symptoms). Subsequent study visits were dependent on whether household member(s) were confirmed positive for SARS‐CoV‐2 at baseline (Figure 1, Table S1). Households in which at least one household member was PCR‐positive for SARS‐CoV‐2 at baseline (‘day 1’) entered into an intensive schedule of follow‐up visits (day 4, 7, 10, 14, 21, and 28 days). If all household members were PCR‐negative for SARS‐CoV‐2 at baseline, they were followed up at day 4 and day 7; if a SARS‐CoV‐2 infection developed within the household, the household entered the intensive follow‐up schedule. If all household members remained PCR‐negative for SARS‐CoV‐2 by day 7 post‐enrolment, the whole household exited the study. In households undergoing the intensive follow‐up schedule, every household member was asked to complete a daily symptom diary. At each visit, naso‐oropharyngeal swabs were taken from all household members and their COVID‐19 vaccination status was recorded. Blood samples were taken on day 7, day 14, and day 28 after identification of the first PCR‐positive within the household (Figure 1, Table S1).

### 2.4 Laboratory methods

The KEMRI‐Wellcome Trust Research Programme Kilifi laboratory was the designated laboratory for testing SARS‐CoV‐2 suspect cases across the coastal region.^17^ Viral RNA was purified using multiple commercial kits including QIAamp Viral RNA extraction Mini Kit, TIANamp Virus RNA kit, SpinX, DAAN Gene Extraction Kit, Qiacube HT method, and Radi Prep Extraction kit. Nucleic acid extract was analysed using commercial SARS‐CoV‐2 RT‐PCR Kits that targeted the Envelope (E) gene, replicase‐encoding region (ORF1ab/RdRp) and Nucleocapsid (N) gene. Necessary diagnostic positive and negative controls were included in each run. Diagnostic results were reported back to the County RRT and to the Ministry of Health (MOH). Results were relayed back to the households by the County RRT.

All serums were separated within 48 h of sample collection and stored at −80°C at the KWTRP laboratories in accordance with Standard Operating Procedures. An in‐house two‐step Enzyme Linked Immunosorbent Assay (ELISA) adapted from the Krammer assay^18^, ^19^ was used to test samples for IgG to SARS‐CoV‐2 whole spike protein. Results were expressed as the ratio of test optical density (OD) to the OD of the plate negative control (a pool of prepandemic sera from 50 Kenyan adults); samples with OD ratios greater than two were considered positive for anti‐SARS‐CoV‐2 IgG. In prior reported validations, sensitivity of the assay was 92.7% (95% CI 87.9%–96.1%); specificity was 99.0% (95% CI 98.1%–99.5%).^19^

### 2.5 Statistical analysis methods

Among households with at least one SARS‐CoV‐2 positive member, participants were defined into three groups: primary cases, secondary cases, and those who remained negative. Primary cases were the earliest confirmed PCR‐positive case(s) within a household at the time of study; secondary cases were household members who were PCR‐negative at the timepoint the primary case(s) in the household were identified but had at least one subsequent PCR‐positive test result during follow‐up; persistently negative household members were defined as those who returned negative PCR results throughout follow‐up (i.e., excluding those lost to follow‐up). Cox regression was used to identify risk factors associated with acquisition, comparing secondary cases with those remaining negative, with robust standard errors to account for clustering by household. Characteristics of participants who were lost to follow‐up were compared with those in the analytical dataset using a generalised linear model with complementary log–log link function with random effects to control for clustering by household.

Secondary attack rates were calculated using the number of secondary cases (defined above) within 7 and 14 days of the identification of the primary case(s), and the number of susceptibles in each household. Susceptibles were household members who were not primary cases, who remained in follow‐up at the timepoint of interest, as we could not be sure that the other susceptibles enrolled, but lost to follow‐up were not cases by this timepoint. Household‐specific attack rates were pooled using binomial regression with a random effect for household (Metaprop).^20^ Secondary attack rates were stratified by the number of primary cases within the household, the household size (total occupants, regardless of the number enrolled in the study), vaccination status of participants, epidemic wave, and the symptom status of the primary case(s).

## 3 ETHICS

The protocol and study forms were approved by the KEMRI Scientific & Ethics Review Unit (SERU; 4077), The University of Warwick's Humanities and Social Sciences Research Ethics Committee (BSREC 150/19‐20) and the London School of Hygiene & Tropical Medicine observational research ethics committee (28160).

## 4 RESULTS

### 4.1 Description of the study population

A total of 214 households consented to participate out of 250 visited. At least one household member in 119 households had a confirmed PCR‐positive result at enrolment, or on day 4 or day 7; these households were therefore followed up intensively for 28 days following the positive test (Figure 1). Households enrolled in intensive follow‐up may have had on average more occupants than those who exited the study because all household members were PCR‐negative at day 7 but were otherwise similar in baseline characteristics (Table S2).

### 4.2 Description of households in intensive follow‐up

Across the 119 households that entered intensive follow‐up, 30% (36/119) were enrolled during the third wave of SARS‐CoV‐2 infection in Kenya (dominated by the Alpha and Beta variants in March–June 2021), another 30% (34/119) were enrolled during wave 4 (predominantly the Delta variant, in July‐Nov 2021), and 40% (48/119) were enrolled in December 2021 to August 2022 during two Omicron variant waves (Table S2). Of 535 household members, 503 (94%) consented to participate in the study, 186 (37%) were PCR‐positive at enrolment, and 234 (46.5%) were seropositive at enrolment (Table S3). Only 483 remained in follow‐up at the visit at which one of their household members first turned PCR‐positive and their household entered intensive follow‐up, 226 (45%) of household members remained in follow‐up at day 14 post‐identification of the primary case (Figure 1). In total, there were 209 primary cases across the 119 households, and after 14 days of intensive follow‐up, 43 secondary cases had been identified and 81 individuals remained negative by RT‐PCR. A further two household members became PCR‐positive 15–28 days after the primary case was identified.

### 4.3 The secondary attack rate

The 7‐day attack rate was restricted to 174 unvaccinated household members who were not defined as primary cases and remained in the study 7 days post‐identification of the primary case (i.e., 64% of 272 enrolled unvaccinated household members who were not a primary case). Accounting for clustering by household, 7 days after identification of the primary case(s), 4% of the unvaccinated household members who had been negative at enrolment had tested positive (95% CI 1%–11%). The 14‐day secondary attack rate was restricted to 113 unvaccinated household members who were not primary cases and remained in the study 14 days post identification of the primary case(s) (i.e., 42% of 272 HH members enrolled who were not a primary case; Table S4). Accounting for clustering by household, 14 days after identification of the primary case(s), 33% of unvaccinated household members who had been negative at enrolment had tested positive (95% CI 17%–40%; Table 1).

**TABLE 1:** Secondary attack rates among unvaccinated participants.

	HHs	Susceptible at 7 days (*N*)	Secondary cases at 7 days (*n*)	SAR^a^ (7 days)	95% CI	Susceptible at 14 days	Secondary cases at 14 days (*n*)	SAR^a^ (14 days)	95% CI
Number of primary cases in the household									
1	65	94	11	0.03	0.00–0.11	65	23	0.31	0.14–0.50
2	29	54	8	0.05	0.00–0.20	37	12	0.35	0.04–0.74
3 or more	16	26	5	0.11	0.00–0.33	11	6	0.45	0.08–0.84
Household size									
2–3 occupants	44	19	5	0.19	0.00–0.49	16	9	0.59	0.25–0.89
4–5 occupants	35	51	7	0.05	0.00–0.18	33	12	0.30	0.09–0.56
6 or more	30	109	13	0.05	0.00–0.12	67	20	0.26	0.08–0.48
Epidemic wave									
2–3 (Alpha/Beta)	34	78	4	0.00	0.00–0.04	43	10	0.17	0.01–0.43
4 (Delta)	32	41	10	0.16	0.03–0.35	31	16	0.55	0.21–0.87
5–6 (Omicron)	43	60	11	0.09	0.01–0.24	42	15	0.32	0.13–0.52
Characteristics of the primary case									
At least one symptom	82	118	16	0.03	0.00–0.10	68	25	0.37	0.17–0.58
Asymptomatic	27	61	9	0.07	0.00–0.19	48	16	0.26	0.08–0.51
Overall	109	174	24	0.04	0.00–0.10	113	41	0.33	0.18–0.49

Abbreviations: CI, confidence interval; HH, household; SAR, secondary attack rate.

^a^ Excludes 11 participants who were vaccinated (two secondary cases and nine susceptible) so the number of secondary cases has decreased from 43 to 41. In a setting with high community transmission, and limited nonpharmaceutical public health interventions the SARs are likely to be overestimated as they will include introduction of new infections into the household from the community. Data are grouped into substrata to avoid sparse data: seven households had three primary cases, five had four primary cases, one had five primary cases, two had six primary cases, and one had seven primary cases. Wald tests for heterogeneity between subgroups from the random effects model for the SAR at 7 (days): by number of primary cases within the household (*p* = 0.66), by household size (*p* = 0.12), by wave (*p* = 0.01), and by symptoms of the primary case (0.81), indicated there was evidence of a difference in SARs at 7 days by wave. Wald tests for heterogeneity between subgroups form the random effects model for the SAR at 14 days: by number of primary cases in the household (*p* = 0.81); by household size (*p* = 0.23) and by epidemic wave (*p* = 0.24), and by symptoms of the primary case (*p* = 0.49) indicated no evidence of a difference in SARs by any of these factors.

Households recruited during the wave of Delta variant infections had higher 7‐day attack rates than prior Alpha/Beta waves and subsequent Omicron waves (*p* = 0.01; Table 1). We found no significant differences between attack rates when stratified by the number of primary cases in the household or whether the primary case(s) reported at least one symptom. When restricted to just households with a single primary case, we found no difference in attack rates by whether the primary case was symptomatic, seropositive, or vaccinated (Table S4). Given the small number of secondary cases per household and the seroprevalence at enrolment among susceptibles (61/117; 52%), we could not perform subanalyses of the secondary attack rate stratified by the serostatus of susceptibles.

### 4.4 The generation time

A total of 33 households had secondary cases that contributed to the calculation of generation time, that is, the time between the primary case's first PCR‐positive result and the secondary case's first PCR‐positive result. We calculated a mean generation time of 6.9 days, median 6 days (IQR 3–9 days, range 3–27). This did not differ when restricted to 30 unvaccinated primary and secondary case pairs (mean 7.3 days, median 6 days [IQR 3–9, range 3–27]).

### 4.5 Characterisation of primary and secondary cases

Over a third of primary cases (46%) only tested positive once (Table 2). Secondary cases generally had short‐lived infections; among those followed up for at least 14 days, 51% had only one PCR‐positive test result, another 34% had their first and last PCR‐positive results 8 days apart. Three secondary cases remained PCR‐positive at visits 24 days apart; there is some indication from genomic data that these may be rapid reinfections with different variants.^21^

**TABLE 2:** Characterisation of cases.

Participant characteristics	Primary cases		Secondary cases within 14 days of primary case			
	*n*	Col %	*n*	Col %	*N*	*p* value^a^
ALL	209		43		252	
**Age**						
0–9 years	38	18.3	15	34.9	53	0.002
10–19 years	43	20.7	5	11.6	48	
20–45 years	85	40.9	8	18.6	93	
>45 years	42	20.2	14	32.6	56	
**Sex**						
Female	137	65.6	26	60.5	163	0.622
Male	72	34.4	17	39.5	89	
**Education**						
None/incomplete primary	97	47.1	26	60.5	123	0.121
Complete primary/incomplete secondary	35	17.0	4	9.3	39	
Compete secondary and above	74	35.9	13	30.2	87	
**Occupation**						
Worker (HCW, teacher, informal, other)	76	36.4	10	22.2	86	0.082
Stays home incl. children, elderly, nonworkers	133	63.6	33	73.3	166	
**Mixing**						
With HH members only	41	19.8	7	16.3	48	0.689
With people outside the household	166	80.2	36	83.7	202	
**Childcare** ^**+**^						
Within the household	107	53.5	22	50	129	0.943
Other	93	46.5	20	45.5	113	
**Smoke at least once a week**						
Yes	1	0.5	1	2.3	2	NR
No	206	99.5	42	97.7	248	
**Any pre‐existing conditions**						
No	178	85.2	38	88.4	216	0.6119
Yes	31	14.8	5	11.6	36	
**Any symptoms in month prior to enrolment**						
Yes	121	57.9	20	46.5	141	0.201
No	88	42.1	23	53.5	111	
**Vaccination status at enrolment**						
At least one dose	21	10.1	2	4.7	23	0.2669
None/unknown	187	89.9	41	95.3	228	
**Serostatus at enrolment**						
Negative	89	50.28	20	47.6	109	0.8529
Positive	88	49.72	20	47.6	108	
**Timing of first PCR+ test**						
Enrolment	186	89.0	0	0	186	NR
V2	17	8.1	20	46.5	37	
V3	5	2.4	11	25.6	16	
V4	1	0.5	11	25.6	12	
V5–9	0	0	1	2.3	1	
**Days between participants' first and last PCR+ test result**						
0	74	46.0	18	51.4	92	NR
3–5	2	1.2	6	17.1	8	
6–8	33	20.5	6	17.1	39	
9–10	20	12.4	1	2.9	21	
13–14	23	14.3	1	2.9	24	
17–24	6	3.7	3	8.6	9	
27–33	3	1.9	0	0.0	3	
**Any symptoms at the first PCR(+) visit, or within 7 days of the PCR(+) visit**						
Yes	123	60.39	8	21.1	122	<0.001
No	81	39.7	30	79.0	120	
**Cumulative seroprevalence relative to participant's first PCR+**						
Before	13	61.9	23	41.8	36	NR
Anytime 0‐7 days on/after their first PCR+	82	68.9	18	60	100	
8–14 days	88	80.7	8	72.7	96	
15–21 days	8	88.9	8	72.7	16	
22–33 days	58	89.2	5	71.4	63	

^a^ *p* values result from likelihood ratio tests of univariable associations between the binary variable of being a primary or secondary case and the risk factor, using a generalised linear model with complementary log–log function, accounting for clustering by household with random effects.

**FIGURE 2:**
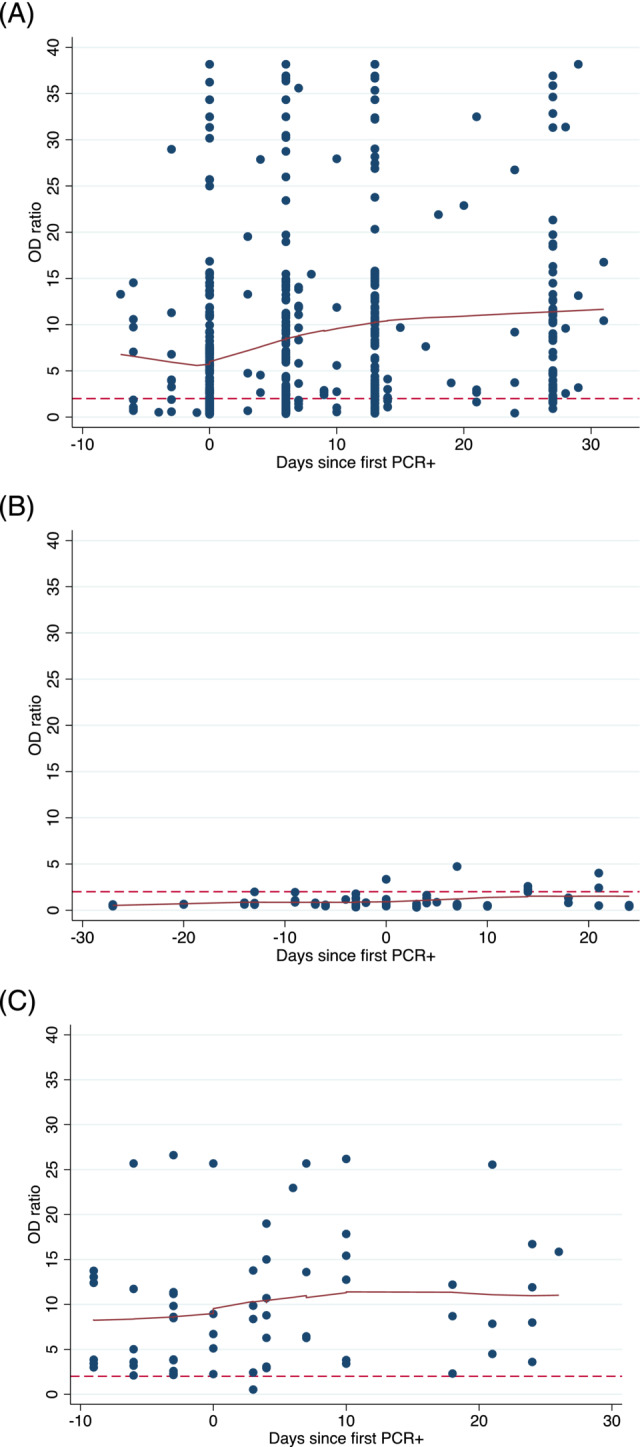
Serological responses over time in (A) primary cases, (B) secondary cases who were seronegative at enrolment, and (C) secondary cases seropositive at enrolment, by time since first PCR+ result. Solid lines result from locally weighted regression model (lowess); red dotted lines indicate the cut‐off of an OD ratio of ‘2’ to define seropositivity.

**FIGURE 3:**
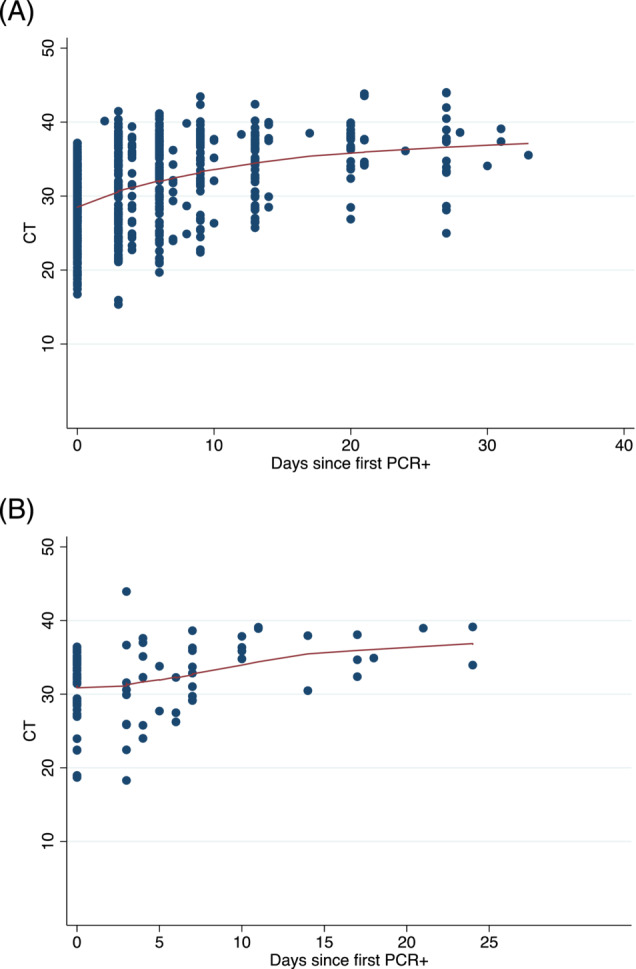
CT values over time in (A) primary cases and (B) secondary cases by time since first PCR+ result. Solid lines result from a locally weighted regression model (lowess).

Half of all the primary and secondary cases were seropositive for IgG to SARS‐CoV‐2 at enrolment (Table 3). Among primary cases and secondary cases, there were small increases in both OD ratios and CT values over time (Figures 2 and 3) indicating increasing serological (IgG) responses and diminishing viral load over time.

**TABLE 3:** Risk factors associated with becoming a secondary case.

Participant characteristics		Secondary cases within 14 days of primary case		Participants who remained negative		Total	HR	Wald *p* value
		*n*	Col %	*n*	Col %			
ALL		43		81		124		
**Age**								
0–9 years		15	34.9	24	29.6	39	1	0.081
10–19 years		5	11.6	20	24.7	25	0.47 (0.16–1.35)	
20–45 years		8	18.6	20	24.7	28	0.64 (0.32–1.29)	
>45 years		14	32.6	17	21.0	31	1.37 (0.69–2.70)	
**Sex**								
Female		26	60.5	53	65.4	79	1	0.629
Male		17	39.5	28	34.6	45	1.14 (0.67–1.95)	
**Education**								
None/incomplete primary		26	60.5	45	55.6	71	1	0.450
Complete primary/incomplete secondary		4	9.3	14	17.3	18	0.53 (0.19–1.43)	
Compete secondary and above		13	30.2	22	27.2	35	0.95 (0.53–1.71)	
**Occupation**								
Worker (HCW, teacher, informal, other)		10	22.2	18	22.2	28	1	0.971
Stays home incl. children, elderly, nonworkers		33	73.3	63	77.8	96	0.99 (0.55–1.79)	
**Mixing**								
With HH members only		7	16.3	14	17.3	21	1	0.789
With people outside the household		36	83.7	67	82.7	103	1.12 (0.49–2.55)	
**Childcare** ^**a**^								
Within the household		22	50.0	42	53.2	64	1	0.849
Other		20	45.5	37	46.8	57	0.94 (0.50–1.76)	
**Smoke at least once a week**								
Yes		1	2.3	2	2.5	3	NR	
No		42	97.7	77	97.5	119		
**Relation to contact**								
The primary case/contact of a case		7	16.3	11	13.6	18	1	0.126
Spouse/child		7	16.3	26	32.1	33	0.37 (0.13–1.04)	
Parent/in‐law/sibling/other		29	67.4	44	54.3	73	0.79 (0.34–1.83)	
**Any pre‐existing conditions**								
No		38	88.4	78	96.3	116	1	0.093
Yes		5	11.6	3	3.7	8	2.19 (0.88–5.44)	
**Any Contact with a confirmed case prior to v1**								
Yes		37	86.0	67	82.7	104	1	0.915
No/unknown		6	14.0	14	17.3	20	0.96 (0.43–2.11)	
**Any symptoms in last month since enrolment**								
Yes		20	46.5	26	32.1	46	1	0.175
No		23	53.5	55	67.9	78	0.65 (0.34–1.21)	
**Vaccination status at enrolment**								
At least one dose		2	4.7	6	7.4	8	1	0.629
None/unknown		41	95.3	75	92.6	116	1.46 (0.32–6.67)	
**Serostatus at enrolment**								
Negative		20	47.6	36	46.8	56	1	0.679
Positive		20	47.6	41	53.2	61	0.88 (0.48–1.61)	

*Note*: Crude hazard ratios and Wald *p* values were produced from a survival analysis of time to becoming a secondary case with censoring of observations when participants became lost to follow‐up or exited the study, using robust standard errors to account for clustering by household. NR: indicates tests not reported due to sparse data (<5 observations in at least one cell).

^a^ Participants were asked if the children of the household were predominantly looked after by someone from the household or by someone/somewhere outside the household.

Just over half of primary cases (56%, 114/204) reported symptoms. A fifth (21%, 8/38) of secondary cases reported symptoms within 7 days of becoming PCR‐positive, 79% remained asymptomatic (95% CI 66%–92%). Six of the symptomatic secondary cases only reported symptoms at one timepoint, one reported symptoms for 3 days, and one reported symptoms for 6 days. A similar proportion of household members who remained PCR‐negative also reported symptoms (18% 14/77; Table 4).

**TABLE 4:** Symptom prevalence.

Symptom	Primary cases *n* = 209			Secondary cases *n* = 43			Negative *n* = 81		
	*n*	*N*	%	*n*	*N*	%	*n*	*N*	%
Any	114	204	55.9	8	38	21.1	14	77	18.2
Fever	26	43	60.5	0	37	0.0	2	77	2.6
Shortness of breath	7	204	3.4	2	37	5.4	0	77	0.0
Pain	29	204	14.2	2	37	5.4	2	77	2.6
Weakness	31	204	15.2	1	37	2.7	2	77	2.6
Diarrhoea	5	204	2.5	0	37	0.0	0	77	0.0
Cough	93	204	45.6	4	37	10.8	10	77	13.0
Nausea/Vomiting	6	204	2.9	0	37	0.0	0	77	0.0
Sore throat	48	204	23.5	1	37	2.7	2	77	2.6
Headache	48	204	23.5	2	37	5.4	2	77	2.6
Runny nose	58	204	28.4	2	37	5.4	5	77	6.5
Confusion	2	204	1.0	0	37	0.0	0	77	0.0
Loss of taste/smell	10	204	4.9	0	37	0.0	0	77	0.0
Other	10	204	4.9	0	37	0.0	2	77	2.6

*Note*: Simple chi‐squared tests indicated some evidence of a difference in the proportion of secondary cases reporting shortness of breath (5%) and the proportion of those negative for SARS‐CoV‐2 reporting shortness of breath (0%, *p* = 0.04). No other statistically significant differences were observed between symptom prevalence in secondary cases and among those remaining negative.

### 4.6 Risk factors for becoming a secondary case

We found no evidence of an association between characteristics such as age, sex, education, occupation, social mixing, pre‐existing conditions, vaccination, or serostatus at enrolment and acquisition of SARS‐CoV‐2 during follow‐up (Table 3).

Given the low retention rate (45%) of participants at day 14, we analysed differences in participant characteristics among those who were included in the risk factor and secondary attack rate analyses and those whose data were missing. Young people and adults (10–45 year olds), participants with reported pre‐existing conditions and unvaccinated individuals were more likely to drop out of follow‐up compared with young children or adults over 45, participants without pre‐existing conditions and the small number of vaccinated participants (Table S5).

## 5 DISCUSSION

We report a low, 4%, 7‐day attack rate within households in rural, coastal Kenya, lower than those reported in other settings in a recent systematic review, although the follow‐up time of other studies is not always apparent.^7^ There were a number of limitations to the analysis conferred by substantial challenges in implementing the study during the pandemic. There were low numbers of households recruited over time due to low volume of routine tests and referrals to the rapid response team; therefore, recruitment was extended from October 2020 to September 2022 across multiple waves of infection and behaviour changes. However, consent rates among those that were engaged were high. The number of co‐primary cases evident at enrolment indicated we may have missed some of the early transmission events within the household; although genomic data among half the swabs indicate different lineages or sublineages that may mean at least some of these were co‐primary cases as a result of community transmission.^21^ Sensitivity analyses of attack rates, restricted to households with just one primary case were very similar to the overall attack rates (Table S4). The study suffered substantial losses to follow‐up and those lost to follow‐up were more likely to be young people and adults of working age, and/or those with co‐morbidities, who were potentially at higher risk of infection.

The 14‐day attack rate of 33% was closer to the global average (30%–43%)^7^ and very similar to the 14‐day attack rate reported in South Africa^13^; however, genomic data on half of the swabs indicates that this includes multiple introductions from outside of the household.^21^ In 34 households, more than one distinct virus was introduced to the household within the 28 days of follow‐up.^21^ Unfortunately, due to the low proportion of swabs that had sufficient nucleic acid to be sequenced (53%), we could not restrict our attack rate analysis to secondary cases with a genomically similar virus to the primary case in the household. We did not find any characteristics that put individuals at significantly higher risk of acquiring SARS‐CoV‐2 during follow‐up, but our analysis was limited by the small sample size and potential misclassification of new introductions as secondary cases.

Our findings that households had a high number of co‐primary cases and high seroprevalence at enrolment, low 7‐day secondary attack rate, and multiple introductions of new genomic variants from outside the household indicate the observed SARS‐CoV‐2 transmission was predominantly community spread, rather than within‐households. Low secondary attack rates within households may be plausible in our context with high seroprevalence after natural exposure to the virus,^22^ heat and UV sunlight reducing virus survival outside the host for subsequent transmission,^23^ and predominantly well‐ventilated outdoor contacts in rural settings.^24^

Natural, vaccine‐induced and hybrid immunity have been shown to both directly protect individuals^25^ and reduce secondary transmission.^6^, ^7^ Half of our participants were seropositive for IgG to SARS‐CoV‐2 at enrolment; this is representative of our setting where seroprevalence in a cross‐sectional community sample in May 2021 was 25%.^22^ We found no evidence of an association between an individual's serostatus and risk of becoming a secondary case; however, symptoms among secondary cases were uncommon and mild.

There were too few participants reporting any one symptom to disaggregate the attack rate by symptom of the primary case. We also could not disaggregate the attack rate by vaccination status as so few of the participants had been vaccinated. This is representative of our context; COVID‐19 vaccination started in Kenya in March 2021, but half of the participants were enrolled before July 2021 when the vaccine coverage with one dose in Kilifi was <5%.^26^ By the end of the study in September 2022, only 17% of adults in Kilifi were fully vaccinated with two doses.^27^

Contrary to a previous review,^7^ we did not see rising secondary attack rates over subsequent epidemic waves; however, the number of households recruited in each wave was small and confidence intervals were wide. The high seroprevalence in our setting indicates a large proportion of the population had already been exposed to SARS‐CoV‐2 by the start of the Omicron waves,^22^ this may have dampened the transmission of this variant but we cannot conclude this from our data.

A total of 30 secondary cases (79%, 95% CI 66%–92%) were completely asymptomatic; this is higher than the 53% estimated in a recent systematic review of studies in Africa.^14^ The prevalence of symptoms and other characteristics in primary cases in our study is likely to be biased, that is, not representative of the prevalence in the general population, due to the reliance on participants presenting to care or to the RRT prior to being enrolled. However, the study follow‐up enabled unbiased detection of secondary cases, where the majority were asymptomatic. The symptoms that were detected were mild and of short duration, and the prevalence of symptoms was not different to those testing negative (Table 4). The low prevalence of symptoms accords with previous observations.^13^, ^28^

## 6 CONCLUSIONS

Households in our setting experienced a lower 7‐day attack rate than a recent meta‐analysis (to March 2022) indicated as the global average. Observed households had a high number of co‐primary cases, high seroprevalence, and high 14‐day attack rate indicating a substantial force of infection in Kilifi, but this may be predominantly via community spread rather than within household transmission. Approximately 80% of secondary cases were asymptomatic.

## AUTHOR CONTRIBUTIONS

**Katherine E. Gallagher:** Conceptualization; data curation; formal analysis; writing—original draft. **Joyce U. Nyiro:** Conceptualization; investigation; project administration; writing—review and editing. **Charles N. Agoti:** Conceptualization; investigation; writing—review and editing. **Eric Maitha:** Investigation. **James Nyagwange:** Investigation; writing—review and editing. **Angela Karani:** Investigation; writing—review and editing. **Christian Bottomley:** Formal analysis; validation; writing—review and editing. **Nickson Murunga:** Data curation; writing—review and editing. **George Githinji:** Investigation; writing—review and editing. **Martin Mutunga:** Data curation; investigation; writing—review and editing. **Lynette Isabella Ochola‐Oyier:** Supervision; writing—review and editing. **Ivy Kombe:** Writing—review and editing. **Amek Nyaguara:** Investigation; writing—review and editing. **E Wangeci Kagucia:** Writing—review and editing. **George Warimwe:** Supervision; writing—review and editing. **Ambrose Agweyu:** Writing—review and editing. **Benjamin Tsofa:** Writing—review and editing. **Philip Bejon:** Writing—review and editing. **J Anthony G Scott:** Writing—review and editing. **David James Nokes:** Supervision; writing—review and editing.

## CONFLICT OF INTEREST STATEMENT

All authors report no conflicts of interest.

### PEER REVIEW

The peer review history for this article is available at https://www.webofscience.com/api/gateway/wos/peer‐review/10.1111/irv.13185.

## ETHICS STATEMENT

The protocol and study forms were approved by the KEMRI Scientific & Ethics Review Unit (SERU; ref 4077), The University of Warwick's Humanities and Social Sciences Research Ethics Committee (HSSREC; ref BSREC 150/19‐20) and the London School of Hygiene & Tropical Medicine observational research ethics committee (ref 28 160).

## PATIENT CONSENT STATEMENT

Written permission to involve the household in the study was sought from the household head. Individual written informed consent was obtained from every household member prior to engagement with any study procedure. Consent for children under the legal age of consent (18 years) was obtained from a parent or legal guardian. Informed assent was obtained from adolescents aged 13–17 years.

## LIST OF MEMBERS OF THE COVID‐19 TESTING TEAM AT KWTRP

Agnes Mutiso, Alfred Mwanzu, Angela Karani, Bonface M. Gichuki, Boniface Karia, Brian Bartilol, Brian Tawa, Calleb Odundo, Caroline Ngetsa, Clement Lewa, Daisy Mugo, David Amadi, David Ireri, Debra Riako, Domtila Kimani, Donwilliams Omuoyo, Edwin Machanja, Elijah Gicheru, Elisha Omer, Faith Gambo, Horace Gumba, Isaac Musungu, James Chemweno, Janet Thoya, Jedida Mwacharo, Jennifer Musyoki, John Gitonga, Johnstone Makale, Justine Getonto, Kelly Ominde, Kelvias Keter, Lydia Nyamako, Margaret Nunah, Martin Mutunga, Metrine Tendwa, Moses Mosobo, Nelson Ouma, Nicole Achieng, Patience Kiyuka, Perpetual Wanjiku, Peter Mwaura, Rita Warui, Robinson Cheruiyot, Salim Mwarumba, Shaban Mwangi, Shadrack Mutua, Susan Njuguna, Victor Osoti, Wesley Cheruiyot, Wilfred Nyamu, Wilson Gumbi, and Yiakon Sein.

## Supporting information


**Table S1:** Visit schedule, including naso‐oropharyngeal swabs and blood draws.
**Table S2:** Household characteristics for the whole study population.
**Table S3:** Household member characteristics across the whole study population, in households that remained negative, and those with at least 1 positive PCR test and therefore followed up intensively for 28 days.
**Table S4:** Secondary Attack Rates additional tables.
**Table S5:** Analysis of whether those lost to follow up differed from those who remained in follow up at day 14 post‐primary case identification.

## Data Availability

Data will be available upon reasonable request to the KEMRI‐Wellcome Trust Research Programme data governance committee (https://dataverse.harvard.edu/dataverse/kwtrp).
