# Supplementary material for: Symptom prevalence and secondary attack rate of SARS‐CoV‐2 in rural Kenyan households: A prospective cohort study
Source: Influenza Other Respir Viruses. 2023 Sep 26;17(9):e13185. doi: 10.1111/irv.13185 (PMC10522480; doi:10.1111/irv.13185)
Supplement: Supplementary file 1 — Table S1: Visit schedule, including naso‐oropharyngeal swabs and blood draws. Table S2: Household characteristics for the whole study population. Table S3: Household member characteristics across the whole study population, in households that remained negative, and those with at least 1 positive PCR test and therefore followed up intensively for 28 days. Table S4: Secondary Attack Rates additional tables. Table S5: Analysis of whether those lost to follow up differed from those who remained in follow up at day 14 post‐primary case identification. [file IRV-17-e13185-s001.docx]

**Suppl. Table 1: Visit schedule, including naso-oropharyngeal swabs and blood draws**

| **Visit schedule for participants^1^** | **V1** | **V2** | **V3** | **V4** | **V5** | **V6** | **V7** | **V8** | **V9** | **Total visits & swabs** | **Total blood draws** |
| --- | --- | --- | --- | --- | --- | --- | --- | --- | --- | --- | --- |
| **If at least one HH member +ve^2^ at D1** | | | | | | | | | | | |
| Visit schedule relative to enrolment (including swabs) | **D1** | **D4** | **D7** | **D10** | **D14** | **D21** | **D28** |  |  | 7 | 4 |
| Visit timing relative to first positive swab sample | (+) | D4 | D7 | D10 | D14 | D21 | D28 |  |  |  |  |
| Blood draws^3^ | b |  | b |  | b |  | b |  |  |  |  |
| **If HH members all D1(-) but at least one is D4(+)^2^** | | | | | | | | | | | |
| Visit schedule relative to enrolment (including swabs) | **D1** | **D4** | **D7** | **D10** | **D13** | **D17** | **D24** | **D31** |  | 8 | 4 |
| Visit timing relative to first positive swab sample | (-) | (+) | D4 | D7 | D10 | D14 | D21 | D28 |  |  |  |
| Blood draws^3^ | b |  |  | b |  | b |  | b |  |  |  |
| **If HH members all D1(-) and D4(-) but at least one is D7(+)^2^** | | | | | | | | | | | |
| Visit schedule relative to enrolment (including swabs) | **D1** | **D4** | **D7** | **D10** | **D13** | **D16** | **D20** | **D27** | **D34** | 9 | 4 |
| Visit timing relative to first positive swab sample | (-) | (-) | (+) | D4 | D7 | D10 | D14 | D21 | D28 |  |  |
| Blood draws^3^ | b |  |  |  | b |  | b |  | b |  |  |
| **If HH members all D1(-), D4(-), D7(-)^2^** | | | | | | | | | | | |
| Visit schedule relative to enrolment (including swabs) | **D1** | **D4** | **D7** |  |  |  |  |  |  | 3 | 1 |
| Visit timing relative to first positive swab sample | (-) | (-) | (-) |  |  |  |  |  |  |  |  |
| Blood draws^3^ | b |  |  |  |  |  |  |  |  |  |  |

^1^ All participants will be enrolled on ‘day 1’ (D1) and asked to complete a daily symptom diary for the duration of their enrolment.

^2^ D1(+) indicates that the nasal and throat swabs analysed with RT-PCR were positive at the visit at ‘day 1’, D1(-) indicates that the result was negative at day 1 etc.

^3^ The blood sample (timing denoted by ‘b’ in the table) volume depends on the age of the participant: 3ml for children aged 1-10 years, 5ml for adolescents and adults above 10 years of age.

**Suppl. Table 2: Household characteristics for the whole study population**

|  | **HH characteristics** | **HHs all negative** | | **HHs with at least 1 positive** | | **Total** | p-value^1^ |
| --- | --- | --- | --- | --- | --- | --- | --- |
|  |  | **n** | **col %** | **n** | **col %** |  |  |
| All HHs in dataset (N) | | 95 |  | 119 |  | 214 |  |
| Enrolment date | |  |  |  |  |  | 0.007 |
|  | wave 1 (Mar-Sept 2020) | 0 | 0.0 | 0 | 0.0 | 0 |  |
|  | wave 2 (Oct 2020- Feb21) | 4 | 4.2 | 1 | 0.8 | 5 |  |
|  | wave 3 (Mar-June 2021) | 47 | 49.5 | 36 | 30.3 | 83 |  |
|  | wave 4 (July - Nov 2021) | 18 | 18.9 | 34 | 28.6 | 52 |  |
|  | wave 5, 6 (Dec 2021 - Sept 2022) | 26 | 27.4 | 48 | 40.3 | 74 |  |
| Nearest Health Facility | |  |  |  |  |  | 0.637 |
|  | KCH | 11 | 11.6 | 16 | 13.4 | 27 |  |
|  | Matsangoni HC | 12 | 12.6 | 18 | 15.1 | 30 |  |
|  | Mavueni | 9 | 9.5 | 8 | 6.7 | 17 |  |
|  | Mnarani Dispensary | 6 | 6.3 | 8 | 6.7 | 14 |  |
|  | Mtondia Dispensary | 21 | 22.1 | 15 | 12.6 | 36 |  |
|  | Pingilikani | 8 | 8.4 | 13 | 10.9 | 21 |  |
|  | OTHERS (<10 at each) | 28 | 29.5 | 41 | 34.5 | 69 |  |
| HH occupants (mean, range) | |  |  |  |  |  |  |
|  | Adults | 2.02 (0-6) | | 2.38 (0-8) | |  | 0.078 |
|  | Children | 1.77 (0-6) | | 2.12 (0-11) | |  | 0.111 |
| No of rooms | |  |  |  |  |  | 0.783 |
|  | 1 | 11 | 11.6 | 19 | 16.0 | 30 |  |
|  | 2 | 20 | 21.1 | 24 | 20.2 | 44 |  |
|  | 3 | 26 | 27.4 | 27 | 22.7 | 53 |  |
|  | 4 | 20 | 21.1 | 22 | 18.5 | 42 |  |
|  | 5 or more | 18 | 18.9 | 27 | 22.7 | 45 |  |
| Av no occupants/ room (mean, range) | | 1.34 (0-5) | | 1.58 (0-7) | |  | 0.074 |
| Av occupants/ bedroom (mean, range) | | 1.87 (0-6) | | 2.26 (0-14) | |  | 0.047 |
| House material | |  |  |  |  |  | 0.648 |
|  | Mud | 33 | 34.7 | 43 | 36.1 | 76 |  |
|  | Cement/ Block walls | 62 | 65.3 | 75 | 63.0 | 137 |  |

^1^Chi2 tests were used to compare proportions; t-tests were used for the difference between two means.

**Suppl. Table 3. Household member characteristics across the whole study population, in households that remained negative, and those with at least 1 positive PCR test and therefore followed up intensively for 28 days**

|  |  | **HHs all negative** | | **HHs with at least 1 positive** | |  |
| --- | --- | --- | --- | --- | --- | --- |
| **HH member characteristics** | | **n** | **col %** | **n** | **col %** | Total |
| **All** |  | 360 |  | 535 |  | 895 |
| HH members enrolled | | 262 | 72.8 | 503 | 94.0 | **765** |
| **Retention (% of enrolled)** | |  |  |  |  |  |
| (Day 4 post enrolment) | | 198 | 76% | 454 | 90% |  |
| (Day 7 post enrolment) | | 132 | 50% | 369 | 74% |  |
| (Day 10 post enrolment) | | 13 |  | 303 | 60% |  |
| (Day 14 post enrolment) | | 6 |  | 242 | 48% |  |
| (Day 21 post enrolment) | | 5 |  | 177 | 35% |  |
| (Day 28 post enrolment) | | 4 |  | 104 | 21% |  |
| **Retention (% of those enrolled)** | |  |  |  |  |  |
| Day of first PCR+ in the HH | |  |  | 483 | 96% |  |
| Day 7 post first PCR+ in the HH | |  |  | 354 | 70% |  |
| Day 14 post first PCR+ in the HH | |  |  | 226 | 45% |  |
| Day 28 post first PCR+ in the HH | |  |  | 88 | 17% |  |
| Age of HH members | |  |  |  |  |  |
|  | 0-9 yrs | 52 | 19.9 | 110 | 22.0 | 162 |
|  | 10-19 yrs | 47 | 18.0 | 134 | 26.7 | 181 |
|  | 20-45 yrs | 99 | 37.9 | 165 | 32.9 | 264 |
|  | 46-65 yrs | 43 | 16.5 | 71 | 14.2 | 114 |
|  | >65yrs | 20 | 7.7 | 21 | 4.2 | 41 |
| Sex |  |  |  |  |  |  |
|  | Female | 160 | 61.1 | 301 | 59.8 | 461 |
|  | Male | 102 | 38.9 | 202 | 40.2 | 304 |
| Highest level of schooling | |  |  |  |  |  |
|  | none | 50 | 19.2 | 95 | 18.9 | 145 |
|  | incomplete primary | 73 | 28.0 | 163 | 32.4 | 236 |
|  | complete primary | 24 | 9.2 | 53 | 10.5 | 77 |
|  | incomplete secondary | 17 | 6.5 | 33 | 6.6 | 50 |
|  | complete secondary and above | 96 | 36.8 | 153 | 30.4 | 249 |
|  | unknown | 1 | 0.4 | 6 | 1.2 | 7 |
| Highest level of schooling among >18 yr olds | |  |  |  |  |  |
|  | none | 27 | 16.1 | 42 | 15.6 | 69 |
|  | incomplete primary | 23 | 13.7 | 30 | 11.1 | 53 |
|  | complete primary | 19 | 11.3 | 43 | 15.9 | 62 |
|  | incomplete secondary | 9 | 5.4 | 10 | 3.7 | 19 |
|  | complete secondary and above | 89 | 53.0 | 139 | 51.5 | 228 |
|  | unknown | 1 | 0.6 | 6 | 2.2 | 7 |
| Occupation | |  |  |  |  |  |
|  | Health worker | 23 | 8.8 | 19 | 3.8 | 42 |
|  | teacher or school worker | 10 | 3.8 | 25 | 5.0 | 35 |
|  | agricultural worker | 11 | 4.2 | 5 | 1.0 | 16 |
|  | informal sector labour | 13 | 5.0 | 25 | 5.0 | 38 |
|  | No work incl. children, elderly, non-workers | 170 | 65.1 | 357 | 71.0 | 527 |
|  | Other (incl student, Business, accountant, driver, banker, supermarket attendant) | 34 | 13.0 | 72 | 14.3 | 106 |
| In the last month participant has been mostly | |  |  |  |  |  |
|  | Inside the home with members of your household | 77 | 29.5 | 84 | 16.7 | 161 |
|  | Inside the home with a mix of household members and other people | 8 | 3.1 | 31 | 6.2 | 39 |
|  | Outside the home with other household members | 14 | 5.4 | 21 | 4.2 | 35 |
|  | Outside the home with a mixture of household members, neighbours or others. | 158 | 60.5 | 363 | 72.2 | 521 |
|  | Other | 4 | 1.5 | 4 | 0.8 | 8 |
| In the last month children have been taken care of by | |  |  |  |  |  |
|  | HH member | 61 | 23.4 | 150 | 29.8 | 211 |
|  | themselves | 52 | 19.9 | 118 | 23.5 | 170 |
|  | Other | 142 | 54.4 | 219 | 43.5 | 361 |
|  | unknown | 6 | 2.3 | 16 | 3.2 | 22 |
| Smoke at least once a week | |  |  |  |  |  |
|  | yes | 3 | 1.2 | 7 | 1.4 | 10 |
|  | No | 255 | 98.8 | 489 | 98.6 | 744 |
| Ethnicity | |  |  |  |  |  |
|  | Giriama | 138 | 52.9 | 245 | 48.7 | 383 |
|  | Chonyi | 55 | 21.1 | 96 | 19.1 | 151 |
|  | Kikuyu | 13 | 5.0 | 23 | 4.6 | 36 |
|  | Kamba | 3 | 1.1 | 17 | 3.4 | 20 |
|  | Luo | 5 | 1.9 | 10 | 2.0 | 15 |
|  | other | 47 | 18.0 | 112 | 22.3 | 159 |
| Relationship to identified index case/contact of a case | |  |  |  |  |  |
|  | the primary case/ contact of a case | 96 | 36.6 | 118 | 23.5 | 214 |
|  | spouse | 25 | 9.5 | 36 | 7.2 | 61 |
|  | parent/in-law | 39 | 14.9 | 110 | 21.9 | 149 |
|  | sibling | 14 | 5.3 | 38 | 7.6 | 52 |
|  | child | 59 | 22.5 | 94 | 18.7 | 153 |
|  | aunt/uncle | 5 | 1.9 | 35 | 7.0 | 40 |
|  | niece/nephew | 1 | 0.4 | 6 | 1.2 | 7 |
|  | other (incl. house help, friends, students) | 23 | 8.8 | 65 | 12.9 | 88 |
|  |  |  |  |  |  |  |
| HH members PCR positive at enrolment | | 0 | 0.0 | 186 | 37.0 | 186 |
| HH members Seropositive at enrolment | | 113 | 43.1 | 234 | 46.5 | 347 |

**Suppl. Table 4: Secondary Attack Rates additional tables**

1. **Participants contributing to the SAR analysis**

|  | **Primary cases** | **PCR-negative** | **Secondary cases** | **Total in follow-up** | **Total in SAR analysis** | **% retention for the SAR^1^** |
| --- | --- | --- | --- | --- | --- | --- |
| **Overall** |  |  |  |  |  |  |
| At time of first PCR+ in the HH | 209 | 274 | 0 | 483 |  |  |
| 7 days post first PCR+ in the HH | 166 | 161 | 27 | 354 | 188 | 69% |
| 14 days post first PCR+ in the HH | 102 | 81 | 43 | 226 | 124 | 45% |
| **Unvaccinated only** |  |  |  |  |  |  |
| At time of first PCR+ in the HH | 188 | 272 | 0 | 460 |  |  |
| 7 days post first PCR+ in the HH | 145 | 150 | 24 | 319 | 174 | 64% |
| 14 days post first PCR+ in the HH | 81 | 72 | 41 | 194 | 113 | 42% |

1. **SARs among all participants (vaccinated and unvaccinated) by number of primary cases in the household**

|  | HHs | Susceptibles at 7 days (N) | Secondary cases at 7 days (n) | **SAR^2^**  **(7 days)** | 95%CI | Susceptibles at 14 days | Secondary cases at 14 days (n) | **SAR^2^**  **(14 days)** | 95%CI |
| --- | --- | --- | --- | --- | --- | --- | --- | --- | --- |
| 1 primary case | 68 | 99 | 13 | **0.04** | 0.00-0.12 | 68 | 24 | **0.29** | 0.15-0.45 |
| 2 primary cases | 31 | 58 | 8 | **0.03** | 0.00-0.14 | 42 | 13 | **0.21** | 0.06-0.40 |
| 3 primary cases | 10 | 10 | 1 | **0.03** | 0.00-0.37 | 5 | 1 | **0.12** | 0.00-0.67 |
| 4 primary cases | 5 | 10 | 4 | **0.37** | 0.03-0.79 | 7 | 4 | **0.63** | 0.15-1.00 |
| 5 primary cases | 2 | 4 | 1 | **~** | ~ | 1 | 1 | **~** |  |
| 6 primary cases | 2 | 2 | 0 | **~** | ~ | 1 | 0 | **~** |  |
| 7 primary cases | 1 | 5 | 0 | **~** | ~ | 0 | 0 | **~** |  |
| Overall | 119 | 188 | **27** | **0.04** | 0.01-0.11 | 124 | 43 | **0.27** | 0.17-0.39 |

1. **among unvaccinated, restricted to HHs with just one primary case**

| Primary case characteristics | HHs | Susceptibles at 7 days (N) | Secondary cases at 7 days (n) | **SAR^2^**  **(7 days)** | 95%CI | Susceptibles at 14 days | Secondary cases at 14 days (n) | **SAR^2^**  **(14 days)** | 95%CI |
| --- | --- | --- | --- | --- | --- | --- | --- | --- | --- |
| at least one symptom | 43 | 53 | 5 | **0.01** | 0.00-0.11 | 32 | 13 | **0.37** | 0.15-0.61 |
| Asymptomatic | 22 | 41 | 6 | **0.08** | 0.00-0.27 | 33 | 10 | **0.24** | 0.02-0.54 |
| Seronegative | 31 | 50 | 3 | **0.00** | 0.00-0.08 | 37 | 8 | **0.12** | 0.00-0.31 |
| Seropositive | 26 | 37 | 6 | **0.08** | 0.00-0.30 | 23 | 13 | **0.59** | 0.26-0.90 |
| Unvaccinated | 61 | 89 | 11 | **0.03** | 0.00-0.12 | 61 | 22 | **0.32** | 0.14-0.52 |
| At least 1-dose | 4 | 5 | 0 | **0.00** | 0.00-0.37 | 4 | 1 | **0.18** | 0.00-0.77 |
| Overall | 65 | 94 | 11 | **0.03** | 0.00-0.11 | 65 | 23 | **0.31** | 0.14-0.50 |

^1^ Primary cases are excluded from the analysis of secondary attack rates so retention is the number of susceptible and secondary cases who remained in follow up, divided by the total susceptible at the timepoint of identification of the first PCR+ in the household

^2^SARs and 95% confidence intervals were produced using binomial regression with random effects for each household (*metaprop*).

The tests for heterogeneity between the SARS at 7-days (p=0.62); and at 14 days (p=0.43) indicate no evidence the SARS significantly differ by the symptom status of the primary case even when restricted to households with only one primary case. Nor by serostatus in the SAR for 7 days (0.26) but come evidence of a difference in SAR at 14 days by serostatus (p=0.05). There was no evidence of a difference in SAR at 7 or 14 days by vaccination status of the primary case (p=0.68, p=0.60 respectively).

**Suppl. Table 5. Analysis of whether those lost to follow up differed from those who remained in follow up at day 14 post-primary case identification**

| **Participant characteristics** | | **Analysis dataset** | | **LTFU** | | **Total** | **HR** | **95%CI** | **p-value**† |
| --- | --- | --- | --- | --- | --- | --- | --- | --- | --- |
|  |  | **n** | **row %** | **n** | **row %** |  |  |  |  |
| ALL |  | **124** |  | **170** |  | **294** |  |  |  |
| **Age** | |  |  |  |  |  |  |  |  |
|  | 0-9 yrs | 39 | 54.2 | 33 | 45.8 | 72 | 1 |  | 0.005 |
|  | 10-19 yrs | 25 | 27.5 | 66 | 72.5 | 91 | 1.93 | 1.09-3.43 |  |
|  | 20-45 yrs | 28 | 35.0 | 52 | 65.0 | 80 | 1.46 | 0.82-2.60 |  |
|  | >45 yrs | 31 | 62.0 | 19 | 38.0 | 50 | 0.64 | 0.31-1.32 |  |
| **Sex** | |  |  |  |  |  |  |  |  |
|  | Female | 79 | 48.2 | 85 | 51.8 | 164 | 1 |  | 0.226 |
|  | Male | 45 | 34.6 | 85 | 65.4 | 130 | 1.30 | 0.85-1.97 |  |
| **Education** | |  |  |  |  |  |  |  |  |
|  | None/ incomplete primary | 71 | 44.1 | 90 | 55.9 | 161 | 1 |  | 0.783 |
|  | complete primary/ incomplete secondary | 18 | 35.3 | 33 | 64.7 | 51 | 1.07 | 0.61-1.90 |  |
|  | compete secondary and above | 35 | 44.3 | 44 | 55.7 | 79 | 0.87 | 0.52-1.46 |  |
| **Occupation** | |  |  |  |  |  |  |  |  |
|  | Worker (HCW, teacher, informal, other) | 28 | 40.0 | 42 | 60.0 | 70 | 1 |  | 0.558 |
|  | Stays home incl. children, elderly, non-workers | 96 | 42.9 | 128 | 57.1 | 224 | 1.16 | 0.70-1.92 |  |
| **Mixing** | |  |  |  |  |  |  |  |  |
|  | with HH members only | 21 | 32.8 | 43 | 67.2 | 64 | 1 |  | 0.343 |
|  | with people outside the household | 103 | 45.2 | 125 | 54.8 | 228 | 0.76 | 0.44-1.33 |  |
| **Childcare** | |  |  |  |  |  |  |  |  |
|  | within the hh | 64 | 39.8 | 97 | 60.3 | 161 | 1 |  | 0.598 |
|  | other | 57 | 45.2 | 69 | 54.8 | 126 | 0.88 | 0.56-1.40 |  |
| **Smoke at least once a week** | |  |  |  |  |  |  |  |  |
|  | yes | 3 | 50.0 | 3 | 50.0 | 6 | 1 |  | 0.876 |
|  | No | 119 | 42.1 | 164 | 58.0 | 283 | 1.09 | 0.22-5.36 |  |
| **Relation to contact** | |  |  |  |  |  |  |  |  |
|  | The primary case/ contact of a case | 18 | 64.3 | 10 | 35.7 | 28 | 1 |  | 0.061 |
|  | Spouse/ child | 33 | 38.8 | 52 | 61.2 | 85 | 2.28 | 0.97-5.33 |  |
|  | Parent/ in-law/ sibling/ other | 73 | 40.3 | 108 | 59.7 | 181 | 2.45 | 1.10-5.46 |  |
| **Any pre-existing conditions** | |  |  |  |  |  |  |  |  |
|  | No | 116 | 44.8 | 143 | 55.2 | 259 | 1 |  | 0.046 |
|  | yes | 8 | 22.9 | 27 | 77.1 | 35 | 2.02 | 1.01-4.04 |  |
| **Any Contact with a confirmed case prior to enrolment** | |  |  |  |  |  |  |  |  |
|  | Yes | 104 | 43.3 | 136 | 56.7 | 240 | 1 |  | 0.214 |
|  | No/ unknown | 20 | 37.0 | 34 | 63.0 | 54 | 1.53 | 0.78-3.01 |  |
| **Any symptoms in last month since enrolment** | |  |  |  |  |  |  |  |  |
|  | Yes | 46 | 45.5 | 55 | 54.5 | 101 | 1 |  | 0.338 |
|  | No | 78 | 40.4 | 115 | 59.6 | 193 | 1.26 | 0.79-2.01 |  |
| **Vaccination status at enrolment** | |  |  |  |  |  |  |  |  |
|  | at least one dose | 8 | 72.7 | 3 | 27.3 | 11 | 1 |  |  |
|  | none/unknown | 116 | 41.3 | 165 | 58.7 | 281 | 3.92 | 0.93-16.5 | 0.035 |
| **Serostatus at enrolment** | |  |  |  |  |  |  |  |  |
|  | Negative | 56 | 42.8 | 75 | 57.3 | 131 | 1 |  | 0.454 |
|  | Positive | 61 | 41.8 | 85 | 58.2 | 146 | 1.19 | 0.75-1.90 |  |

† crude hazard ratios and LRT p-values were produced from a generalized linear model with a complementary log-log link function, comparing 124 participants in the risk factor analysis at day 14 to the 170 participants who were lost to follow up by this point, using random effects to account for clustering by household. Primary cases (n=209) were excluded from this analysis.
